# Exclusive breastfeeding and rotavirus vaccination are associated with decreased diarrheal morbidity among under-five children in Bahir Dar, northwest Ethiopia

**DOI:** 10.1186/s40985-018-0107-6

**Published:** 2018-11-01

**Authors:** Ghion Shumetie, Molla Gedefaw, Adane Kebede, Terefe Derso

**Affiliations:** 1 ORDA/PSI MULU HIV Prevention Project, Bahir Dar, Amhara region Ethiopia; 2 College of Medicine and Health Science, Bahir Dar University, Bahir Dar, Ethiopia; 3 Department of Health Service Management and Heath Economics, Institute of Public Health, College of Medicine and Health Sciences, University of Gondar, Gondar, Ethiopia; 4 Department of Human Nutrition, Institute of Public Health, College of Medicine and Health Sciences, University of Gondar, Gondar, Ethiopia

**Keywords:** Diarrheal morbidity, Rotavirus vaccination, Under five, Ethiopia

## Abstract

**Background:**

More than one in every ten (14%) of under-five child deaths is associated with diarrheal morbidity in Ethiopia. Although Ethiopia has implemented different health interventions like its immunization program, childhood diarrhea morbidity, on which literature is limited, continues as a public health problem. Hence, the aim of this study was to assess the prevalence of diarrheal morbidity and associated factors among under-five children in Bahir Dar, northwest Ethiopia.

**Method:**

A community based cross-sectional study was carried out from March 05 to April 03/2015 in Bahir Dar in which 553 mother-child pairs participated. A structured questionnaire was adapted from the World Health Organization (WHO) and the Ethiopian Demography and Health Survey (EDHS) to collect the data. Bivariate and multivariate logistic regression analyses were carried out to identify the independent predictors of diarrheal morbidity.

**Result:**

The overall prevalence of diarrheal morbidity was 9.4% [95% Confidence Interval (CI): 4.8, 14.0%]. No receipt of Rotavirus vaccine dose 2 [AOR = 3.96, 95%CI; 2.13, 7.33], non-exclusive breastfeeding [AOR = 2.69, 95%CI; 1.39, 5.19], unavailability of solid waste disposal system [AOR = 2.62, 95%CI; 1.19, 5.77], employed and private business occupational status of mothers [AOR = 2.10, 95%CI; 1.02, 4.31)], and less than Ethiopia Birr (ETB) 600 household monthly income [AOR = 2.10, 95% CI; 1.2, 7.2] were independently associated with diarrheal morbidity.

**Conclusion:**

In Bahir Dar, one in every ten of the under-five children surveyed suffered from diarrheal morbidity. Thus, implementing effective rotavirus vaccination programs, encouraging exclusive breastfeeding and emphasizing appropriate solid waste management would reduce childhood diarrheal morbidity in the region. In addition, the finding suggests that improved child care mechanisms, especially for mothers working outside the home, and efforts to increase household income should be intensified to reduce incidence of diarrhea.

## Background

Diarrhea is associated with many common childhood infectious diseases and one of the immediate causes of undernutrition, which in turn interferes with physical growth, mental development, and increases the risk of death [1, 2]. It remains the second leading cause of death in children under 5 years of age, and 90% of the burden is in resource limited settings [1, 3]. About 50% of childhood morbidity and 50–80% of childhood mortality is associated with diarrhea in Sub-Saharan Africa [4, 5]. Each year, an estimated 2.5 billion cases of diarrhea occur among under-five children, but the incidence has been relatively stable over the past two decades [6]. In resource limited countries, poor declining trend of diarrheal disease is associated with multiple reasons such as, poor environmental sanitation, low educational status of mothers, and other behavioral issues [7, 8].

A variety of bacterial, viral, and parasitic organisms are able to spread through contaminated food or drinking water or from person to person as a result of poor hygiene [2]. Rotavirus in particular is the most common etiological agent of diarrhea both in high and low income countries, and 6% of all child death globally is associated with it [9, 10], and child diarrheal morbidity mostly depends on the interaction of behavioral, socio-economic, and environmental factors [11, 12]. Various studies in different settings have explored that unavailability of water, lack of hand washing facilities, hand washing with water only, private business, children aged between 6 and 24 months, illiteracy of mothers, delay to initiate breastfeeding, no breastfeeding, and lack of exclusive breastfeeding were positively associated with diarrheal morbidity [13–16]. On the other hand, rotavirus vaccination has a protective effect on diarrheal morbidity [16].

Ethiopia is implementing health strategies [17, 18] and immunization programs, including more recently rotavirus vaccination, to prevent the burden of diarrheal morbidity [19]. However, about 21% of under-five children are still suffering from diarrhea due to rotavirus [20]. Also, more than one in every ten (14%) of under-five child deaths is associated with diarrhea morbidity in the country [21]. According to the Ethiopian Demographic and Health Survey, the prevalence of diarrheal disease in under-five children in the 2 weeks before the survey has dropped from 18% in 2005 to 13% in 2011 [22, 23]. Conducting a study in an evidence dearth setting is critical to explore information on diarrheal morbidity and its determinants. The study is expected to provide a prominent input to policymakers and program managers about the implementation of current strategies, including rotavirus vaccination. Therefore, the aim of this study was to assess the prevalence and associated factors of diarrheal morbidity among under-five children in Bahir Dar, northwest Ethiopia.

## Methods

### Study setting and design

**Fig. 1:**
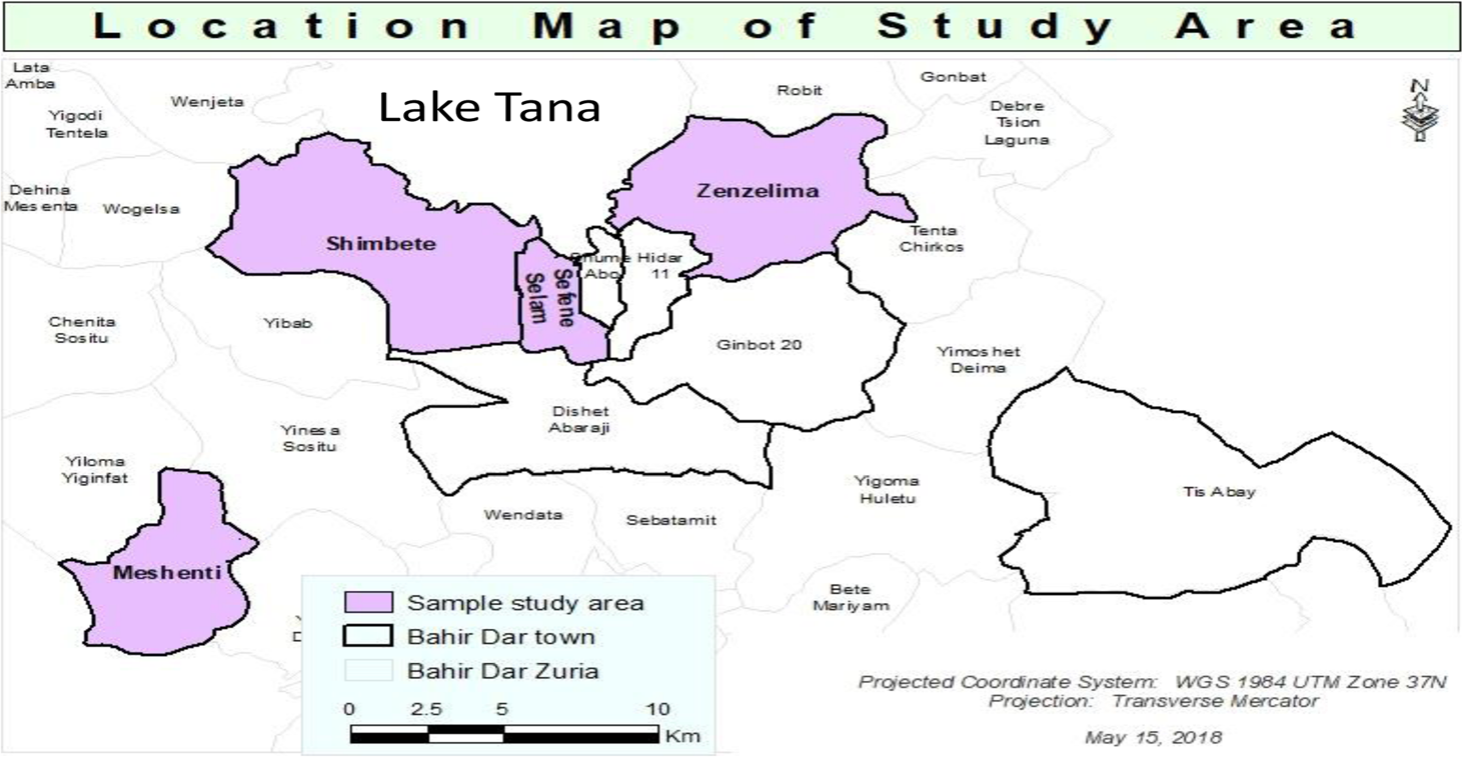
Presentation of the study area. NB: Bahir Dar Zuria is one district in west Gojjam which surround Bahir Dar town

A community based cross-sectional study was carried out from March 05 to April 03, 2015 in Bahir Dar, the capital of Amhara national regional state, which has an estimated population of 297,749 [24]. Bahir Dar is located 565 km from Addis Ababa in Amhara national regional state in northwest Ethiopia. The major economic sectors of the city are horticulture, commerce, agro-industrial processing, urban agriculture, manufacturing, and diverse service industries. Bahir Dar is also one of the leading tourist destinations in Ethiopia; attractions include the nearby Lake Tana and Blue Nile river. Currently, Bahir Dar is divided into 19, 9 urban and 10 rural kebeles (smallest administrative unit). According to the zonal health department, the city administration has 1 referral hospital, 1 zonal governmental hospital, 10 government health centers, 10 health posts, one private hospital, 10 advanced and 17 medium clinics, and 12 small private clinics which provide comprehensive health services including vaccination. Four, 2 urban and 2 rural kebeles (Zenzelima, Sefen Selam, Shimbete and Meshenti) were selected out of 19 kebeles by lottery method (Fig. 1).

### Study population and sampling procedure

All mothers/caregivers with under-five children who lived in Bahir Dar for at least 6 months were included in this study**.** Sample size was calculated using the single population proportion formula on the following assumptions: 95% confidence interval, 5% margin of error, prevalence of diarrhea 21.5% in Jabithennan district, northwest Ethiopia [25], 10% non-response rate, and a design effect of 2%. The final calculated sample size was 572. A multistage sampling technique was employed to select participants; initially, the list of the dwellers of the 19 kebeles was taken from the city administration. Four kebeles were selected using the lottery method. Then, the computed sample size was proportionally allocated according to the number of under-five children in each kebele. Finally, a systematic sampling technique was employed to obtain the required study participants. In cases of more than one study child in a household, the simple random sampling method was used to select a single participant. The outcome variable, diarrhea morbidity, is the presence of three and more loose or liquid stools per day within the 2-week period prior to survey.

### Data collection tools and procedures

A structured questionnaire was adapted from the World Health Organization (WHO) and the Ethiopian Demography and Health Survey (EDHS) to collect the data [23, 26]. The questionnaire was composed of socio-demographic, maternal and child characteristics, child feeding practices, environmental health conditions, and the outcome interest (diarrhea morbidity). Eight diploma graduate and four BSc nurses were recruited for data collection and supervision, respectively. One-day training was given to data collectors and supervisors. A pretest was administered out of the actual study area. After a few modifications like clarifying the ambiguity of word expressions, the questionnaire was administered in the local language, Amharic.

### Data analysis

Data were entered into the EPI-info version7 statistical software and exported to SPSS version 20 statistical package for analysis. Frequencies and proportions were used to summarize variables in tables. The association between diarrhea morbidity and each independent variable was assessed using the binary logistic regression model. Variables significant at *P* value < 0.2 in the bivariate analysis were further entered into the multivariate analysis. The significance of association was determined at a *P* value of< 0.05 in the multivariate analysis, while the strength of association was measured by adjusted odds ratio with 95% confidence interval.

## Results

### Socio-demographic and behavioral characteristics of study participants

In this study, 553 eligible under five years children were included with a response rate of 96.7%. More than three-fourths of the participating mothers (80.5%) were married, and 83.2% had primary school and above educational status. The majority of the mothers (61.7%) were employed or in private business with 41.6% of their children ranging in age from 7 to 24 months.

Nine in every ten (91.7%) of the households had latrine facilities. However, 71.1% had no hand washing facilities near toilets. Three fourths (76.9%) of the participants had a habit of hand washing after visiting toilets. However, more than half (57.9%) of the participants were practiced hand washing without using detergents. Two-thirds of children were vaccinated with rotavirus vaccine dose 1 (66.5%) and dose 2 (64.0%). 71.6% of under-five children were exclusively breastfed for 6 months (Table 1).

**Table 1:** Socio-demographic and behavioral characteristics of study participants in Bahir Dar city administration, northwest Ethiopia, 2015

Characteristics	Frequency	Percentage (%)
Age of mothers in years		
15–24	186	33.6
25–35	292	52.8
> 35	75	13.6
Current marital status		
Married	445	80.5
Unmarried	108	19.5
Religion		
Orthodox	403	72.9
Muslim	100	18.1
Protestant and Catholic	50	9.0
Age of child		
0–6 months	105	19.0
7–24 months	230	41.6
> 24 months	218	39.4
Sex of child		
Male	283	51.2
Female	270	48.8
Monthly income of household		
≤ 600	221	40.0
601–2000	156	28.2
≥ 2001	176	31.8
Mothers educational status		
Uneducated	93	16.8
Educated	460	83.2
Mothers occupation		
Housewife	212	38.3
Employed and private business	341	61.7
Latrine availability		
No	46	8.3
Yes	507	91.7
Sanitation facilities		
Improved sanitation facilities*	231	41.77
Unimproved sanitation facilities**	322	58.23
Availability of solid waste facility		
No	110	19.9
Yes	443	80.1
Hand washing facility near toilet		
No	393	71.1
Yes	160	28.9
Availability of liquid waste facility		
No	371	67.1
Yes	182	32.9
Source of water		
Improved drinking water sources^1^	455	82.2
Unimproved drinking water sources^2^	98	17.8
Protected spring water	1	.2
Unprotected well, spring and surface water	32	5.8
Trend of using toilet		
Always	465	84.1
Rarely	46	8.3
Not at all	42	7.6
Hand wash practice		
No	128	23.1
Yes	425	76.9
Rotavirus vaccine dose 1		
No	185	33.5
Yes	368	66.5
Rotavirus vaccine dose 2		
No	199	36.0
Yes	354	64.0
Antenatal care		
No	99	17.9
Yes	454	82.1
Exclusive breastfeeding		
No	157	28.4
Yes	396	71.6
Current breastfeeding status		
Breast milk only	92	16.6
Partially	327	59.1
No breast feed currently	134	24.2

*Pour-flush latrine, ventilated improved pit latrine, **Open pit latrine, communal and open filed

^1^Pipe, protected well and protected spring water a, ^2^Unprotected well, spring and surface water

### Prevalence of diarrhea

Approximately, one in every ten (9.4%) (95% CI: 4.8, 14.0) of the under-five children had diarrheal morbidity in the 2 weeks preceding the survey.

### Factors associated with diarrheal morbidity

The odds of diarrheal morbidity was higher among children who were not exclusively breastfed for 6 months [AOR = 2.69, 95% CI; 1.39, 5.19], and who had no receipt of rotavirus vaccine dose 2 [AOR = 3.96, 95% CI; 2.13, 7.33] compared to children who were exclusively breastfed for 6 months and with receipt of rotavirus vaccine dose 2, respectively. Children from households that had no solid waste disposal systems [AOR = 2.62, 95% CI; 1.19, 5.77] were more likely to develop diarrheal morbidity compared to children from households with availability of solid waste disposal systems. Employed and private business occupational status of mothers [AOR = 2.10, 95%CI; 1.02, 4.31] and ≤ ETB 600 monthly income of households [AOR = 2.10, 95% CI; 1.2, 7.2] were significantly associated with diarrheal morbidity as compared to their counterparts (Table 2).

**Table 2:** Predictors of diarrheal morbidity in Bahir Dar town administration, northwest Ethiopia, May, 2015

Explanatory variable	Diarrheal morbidity Yes No		Crude odds ratio(95% CI)	Adjusted odds ratio(95% CI)
Sex of the child				
Male	33	250	1.74 (0.97, 3.15)	1.65 (0.89, 3.05)
Female	19	251	1.00	1.00
Solid waste disposal system				
No	15	95	1.73 (0.91, 3.23)	2.62 (1.19, 5.77)
Yes	37	406	1.00	1.00
Occupation of the mothers				
House wife	18	194	1.00	1.00
Employed and private business	34	307	1.19 (0.66,2.17)	2.10 (1.02, 4.31)
Monthly income of the family				
≤ 600	26	195	2.47 (1.13, 5.43)	2.99 (1.23, 7.24)
601–2000	9	139	2.27 (0.96, 5.25)	2.65 (0.99, 6.67)
≥ 2001	52	167	1.00	1.00
Rotavirus vaccine dose 2				
No	32	167	3.20 (1.78,5.77)	3.96 (2.13, 7.33)
Yes	20	334	1.00	1.00
Exclusive breastfeeding				
No	22	135	1.20 (1.11, 3.57)	2.69 (1.39, 5.19)
Yes	30	266	1.00	1.00

Hosmer and Lemeshow goodness of fit test was checked; the result was 0.76

## Discussion

In the current study, approximately one in every ten (9.4%) (95% CI: 4.8, 14.0) of under-five children suffered from diarrheal morbidity; children with no receipt of rotavirus vaccine dose 2, no exclusive breastfeeding, unavailability of a solid waste disposal system, employed and private business occupational status of mothers, and household monthly income less than ETB 2000 were independent predictors of diarrheal morbidity.

The prevalence of diarrheal morbidity was similar to the national report of 13% [23] and other local studies in hot spot districts of the Amhara region (13.5%) [27], Hawassa (11.7%) [28], and Jigjiga town (14.6%) [13]. However, the current finding was lower than reported from Hadaleala district, Afar region, Ethiopia (31.3%) [16]. The high prevalence in Afar region compared to the current study might be due to the nomadic nature of the population. Nomads may not have access to basic health care, water, and sanitation services due to their migration from place to place in search of pasture and water. Nomads have no permanent residence and practice open defecation. The main sources of water are rivers, streams, and wells; hence, they are prone to contamination and diarrheal diseases, especially children who routinely play in the unhygienic environment [29].

This study found that children who did not receive rotavirus vaccine dose 2 suffered 3.96 times more from diarrheal morbidity compared to those in receipt of rotavirus vaccine dose 2. A similar local study on children who did not receive rotavirus vaccine dose 2 showed that children suffered from diarrheal morbidity [16]. This might be due to the fact that rotavirus vaccinated children are immunized from the highest impact of acute gastroenteritis (AGE) morbidity in Africa, where the burden of disease is the greatest [30]. This implies that rotavirus vaccination is one of the best ways to prevent diarrheal morbidity and its consequences. Thus, two-dose rotavirus vaccines (dose 1 and 2) should be given for children as part of a comprehensive approach to control diarrhea.

The odds of diarrheal morbidity were 2.69 times higher among under-five children who were not exclusively breastfed for 6 months. A similar finding was observed in nomadic populations of Ethiopia; children who were not exclusively breastfed suffered from diarrheal morbidity [16]. Breast milk contains all the nutrients that an infant needs in the first 6 months of life. Additionally, breast milk contains bioactive factors that augment infants’ immature immune system, providing protection against infection. Also, breast milk is available all the time and is practically free from pathogenic microorganisms. On the other hand, non-exclusive breastfeeding is an important risk factor of infant diseases like diarrheal morbidity. This finding suggest that exclusive breastfeeding during the first 6 months of life is one of the most effective interventions to improve child health [31].

Like the local study from Debrebirehan town [15], the current study found that employed and private business occupational status of mothers was significantly associated with diarrheal morbidity in under-five children compared to housewife mothers occupational status. The possible explanation might be that mothers working outside the home may have less time to better care and feed their children compared to housewives. Another possible reason might be that mothers working outside the home may not have as much contact time to breastfeed their children as compared with housewives.

According to this study, children from households that had no solid waste disposal systems were more likely to develop diarrheal diseases. This study is consistent with another local study in northwest Ethiopia [32]. This might be due to children putting contaminated fingers, pica, or fomites into their mouths while crawling or playing around contaminated environments associated with poor waste disposal practices [29, 33].

The last significant variable, the odds of diarrheal disease, is nearly three times higher among children whose household income is ≤ ETB 600 compared to children whose household income is ≥ ETB 2000. It may be the case that mothers/caregivers who have higher incomes may have the opportunity to buy and use detergents for hand washing and the resources to construct and use standard toilets.

### Limitation

This study showed the prevalence of diarrheal morbidity and its associated factors; like rotavirus vaccination among the most vulnerable population groups, under-five children, in Bahir Dar, Ethiopia where there is scarcity of literature. However, the study has some limitations. For example, the cross-sectional design of the study may limit its capacity to measure the cause-effect relationship between the outcome and the potential correlates. As well, there may be recall bias of diarrhea episodes/rotavirus vaccination and difficulty distinguishing the time order of exposures. Finally, wealth index is not addressed in this study.

## Conclusion

In the study area, approximately one in every ten of the under-five children had diarrheal morbidity. Thus, implementing effective rotavirus vaccination programs, encouraging exclusive breastfeeding, and emphasize appropriate solid waste management would reduce childhood diarrheal morbidity in the region. In addition, the finding suggests that improved child care mechanisms, especially for mothers working outside the home, and efforts to increase household income should be intensified to reduce incidence of diarrhea.

## Abbreviations

AOR: Adjusted odds ratio
CI: Confidence interval
COR: Crude odds ratio
DDS: Dietary diversity score
EDHS: Ethiopian Demographic Health Survey
GDP: Gross Domestic Production
SPSS: Statistical Package for Social Science
VIPL: Ventilated and improved pit latrine
WHO: World Health Organization
